# Perimortem cesarean delivery and subsequent emergency hysterectomy: new strategy for maternal cardiac arrest

**DOI:** 10.1002/ams2.301

**Published:** 2017-08-17

**Authors:** Mayako Goto, Hiroaki Watanabe, Kazuhide Ogita, Tetsuya Matsuoka

**Affiliations:** ^1^ Sensyu Regional Medical Center for Woman's and Children's Health Rinku General Medical Center Izumisano Osaka Japan; ^2^ Department of Acute Care Surgery Shimane University Faculty of Medicine Izumo Simane Japan; ^3^ Rinku General Medical Center Senshu Trauma and Critical Care Center Izumisano Osaka Japan

**Keywords:** Maternal cardiac arrest, perimortem cesarean delivery, pregnant woman, resuscitation, VA‐ECMO

## Abstract

**Cases:**

Perimortem cesarean delivery (PMCD) is the only way to resuscitate pregnant women in cardiac arrest, and has been found to increase maternal resuscitation rate by increasing circulating plasma volume. However, many obstetricians have not experienced a case of PMCD, as situations requiring it are rare. We report our strategy for cases of maternal cardiac arrest, on the basis of a review of published work, and present two case reports from our medical center.

**Outcomes:**

In case 1, PMCD led to death by massive bleeding. In case 2, PMCD and hysterectomy were carried out after the introduction of venoarterial extracorporeal membrane oxygenation, and both mother and baby survived.

**Conclusion:**

We find that rapid hysterectomy as a damage control surgery following PMCD can be life‐saving for both mother and baby.

## Introduction

American Heart Association guidelines for cardiopulmonary arrest (CPA) suggest that perimortem cesarean delivery (PMCD) is the only way to resuscitate pregnant woman in cardiac arrest.^1^ Perimortem cesarean delivery has been found to increase the maternal resuscitation rate by effecting expulsion of the baby, increasing the circulating plasma volume.^2^, ^3^ It is reported that CPA during pregnancy occurs at a rate of 1/30,000.^4^ Although it is extremely rare to meet with this special condition in a patient, it is important to understand what to do for pregnant patients with CPA if it does occur.

We experienced two PMCDs from April 2013 to March 2014. In this study, we report our experience with PMCD and our strategy, along with a review of published works.

## Cases

### Case 1

**Figure 1:**
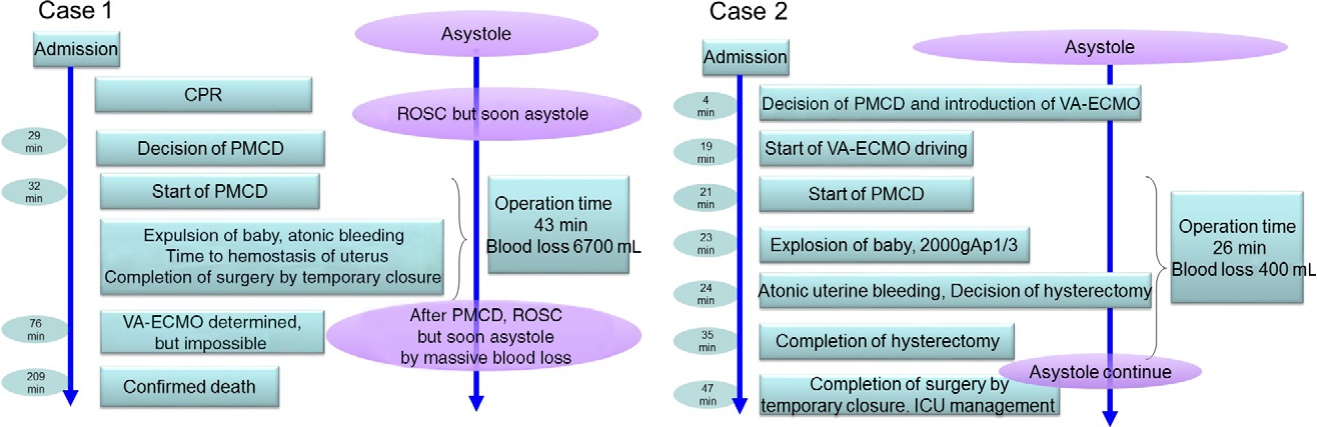
Emergency room processes in two cases of maternal cardiopulmonary arrest. Ap1/3, Apgar score 1/3; CPR, cardiopulmonary resuscitation; PMCD, perimortem cesarean delivery; ROSC, return of spontaneous circulation; VA‐ECMO, venoarterial extracorporeal membrane oxygenation.

The patient was a 33‐year‐old woman (gravida 3, para 2), who had no notable medical history. She consulted her obstetric practitioner for the main complaint of fever and coughing at 37 weeks and was admitted. The patient's respiratory condition became worse, and a disturbance of consciousness was observed; therefore, she was transferred by ambulance to our critical care medical center. She went into CPA in the ambulance, and arrived at our center 9 min after CPA; electrocardiogram showed asystole, and no fetal heart rate was observed. Return of spontaneous circulation was temporarily observed after the patient was admitted, but the hemodynamics were unstable. Twenty‐nine minutes after arrival, it was determined to carry out PMCD. It was difficult to achieve hemostasis due to atonic bleeding and coagulopathy, and therefore took 43 min to operate. We tried to introduce venoarterial extracorporeal membrane oxygenation (VA‐ECMO) after PMCD, but cannulation failed due to narrow blood vessel diameter. The patient died in the emergency room 3 h 29 min after admission (Fig. 1).

### Case 2

The patient was a 29‐year‐old woman (primipara), who also had no unusual medical history. She was admitted due to preterm labor and unexplained hypokalemia. At 36 weeks, she fell into CPA with sudden spasm‐like seizures, and was transferred to our center. She had ventricular fibrillation in the ambulance, and electrocardiogram showed asystole at arrival, which was 30 min after onset of CPA. Fetal heart rate was observed by ultrasound at the time. The decision for PMCD was made 3 min after the patient's arrival in the emergency room. Introduction of VA‐ECMO was completed after 19 min, and PMCD started after 21 min. A baby weighing 2,000 g was delivered after 23 min, with Apgar score 1/3. Because bleeding from the uterus could not be controlled, we quickly performed hysterectomy subsequent to PMCD. Damage control surgery (DCS) was decided on for hemostasis. Gauze was packed into the abdominal cavity and a temporary abdominal closure was carried out using vacuum‐assisted closure. As the patient had a severe coagulopathy, massive transfusion was undertaken in the intensive care unit. A definitive abdominal closure was carried out on the 5th postoperative day, and withdrawal from VA‐ECMO was completed on the 9th postoperative day. Despite sequelae in the nervous system, respiratory support was terminated, and the patient was transferred to a rehabilitation hospital, followed by home discharge of patient and baby (Fig. 1).

## Discussion

General cardiopulmonary resuscitation has been established as a procedure since 1961,^5^ and maternal resuscitation since approximately 1990.^6^ The American Heart Association, the European Resuscitation Council, and the Royal College of Obstetricians and Gynaecologists have published evidence‐based guidelines for perinatal resuscitation.^1^, ^7^, ^8^ It is necessary to modify common resuscitation guidelines for the perinatal context because of maternal physical and anatomical changes. The guidelines of both the International Consensus Conference on Cardiopulmonary Resuscitation and the Emergency Cardiovascular Care Science with Treatment Recommendations have recommended that when normal CPR is not successful in cases of CPA in women more than 20 weeks pregnant, the PMCD decision should be made within 4 min from CPA, and the baby removed within 5 min.^1^, ^7^, ^8^ This principle has also been reflected since 2014 in Japanese practice guidelines. The major issues in case 1 were that it took too long for the PMCD decision to be made and that bleeding due to atonic uterus could not be controlled. Based on this outcome, we adopted a strategy to achieve rapid, safe PMCD, as follows:

1. Induce VA‐ECMO in order to stabilize the circulation;
2. Do the cesarean section immediately after VA‐ECMO introduction;
3. Perform DCS for control of bleeding, if severe coagulopathy is shown.

Stabilization of circulation should be the priority. In particular, brain resuscitation should be prioritized to maintain cerebral blood flow in the mother. If the cause of the bleeding is due to coagulopathy, sometimes hemostasis cannot be achieved surgically. In cases of severe coagulopathy, instead of the usual radical operation, DCS should be carried out. Under CPA, uterine atonicity can naturally occur, and massive bleeding at this time can make resuscitation difficult. In this case, a hysterectomy should be quickly carried out as a tactic to achieve hemostasis. Intra‐abdominal gauze‐packing to promote hemostasis and temporary abdominal closure are an effective strategy for hemostasis and shortening surgery time.

In case 2, the above strategy was implemented based on the experience of case 1. As a result, the PMCD decision time dropped from 29 min to 4 min, and surgical time was reduced from 43 min to 20 min. The amount of bleeding showed an impressive decrease to 400 mL from 6,700 mL. As the decision of hysterectomy and DCS was quick in case 2, bleeding was relatively little. It is suggested that this strategy led not only to reduced surgery time but also to less bleeding.

Two papers by Katz *et al*. have recommended PMCD within 4 min of cardiac arrest.^2^, ^3^ The first paper is a review of PMCD cases from 1875 to 1985; because many of these date from before the establishment of CPR as a standard medical procedure, the majority of them did not involve basic CPR.^3^ The second paper reviews cases of PMCD from 1986 to 2004.^2^ However, time from arrest to PMCD was described only for surviving babies.^2^ In other words, the 4‐min rule originally focused only on outcomes for babies, and therefore, there is no evidence with respect to maternal resuscitation.^9^ Einav *et al*.^9^ examined cases from 1980 to 2010 in order to verify the 4‐min rule. Of 94 CPA cases considered, PMCD was carried out in 87.2% (*n* = 86), but was performed within 4 min in only four cases, with an average time of 16.6 ± 12.5 min from arrest until delivery. This survey showed that it is generally difficult to achieve PMCD within 4 min, unless the mother is in a special environment such as an operating room or intensive care unit at the time of arrest, and that it is possible to improve the prognosis of the mother by performing PMCD within 10 min. In this context, it is necessary to establish a system for progressive to PMCD from cardiac arrest within 10 min for a good prognosis.

**Figure 2:**
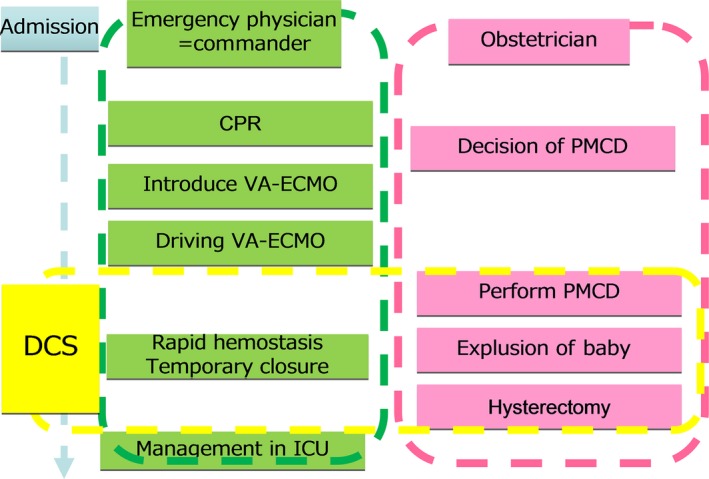
System of perimortem cesarean delivery (PMCD) in our hospital. CPR, cardiopulmonary resuscitation; DCS, damage control surgery; ICU, intensive care unit; PMCD, perimortem cesarean delivery; VA‐ECMO, venoarterial extracorporeal membrane oxygenation.

In Japan, only six cases of PMCD have been reported, including ours (Table 1). Of the six cases, resuscitation was successful in four (67%), and survival to hospital discharge occurred in three (50%). In three cases of successful discharge, two cases of CPA occurred in the operating room just before PMCD. Although one case of successful discharge (our case 2) occurred in the prehospital phase, this case was rescued by stabilization of circulation using VA‐ECMO before PMCD. Damage control surgery in PMCD means speedy hemostasis by abbreviated surgery including hysterectomy and then rapidly starting intensive care. Regarding DCS in cases of PMCD, two recommendations are made on the basis of the results of our experiments: (i) if there is uncontrollable bleeding, quick hysterectomy should be performed for hemostasis; (ii) the operation should be speedily finished using temporary abdominal closure while preventing abdominal compartment syndrome.^10^ Atonic uterine bleeding in a cardiac arrest situation is fatal, and we faced difficulties related to it; therefore, the decision for DCS should be made without hesitation to save the patient's life. Our PMCD system is summarized in Figure 2. The emergency physician, as team leader or person in charge, obstetricians, pediatricians, and co‐medical personnel are included in the team for PMCD. The recommended procedure is:

1. CPR (basic life support and advanced life support) by the emergency physician;
2. VA‐ECMO induction by the emergency physician;
3. Decision for PMCD made by the obstetricians and team leader;
4. PMCD;
5. Rapid hemostasis including hysterectomy and DCS;
6. Intensive care.

**Table 1:** Six reported cases of perimortem cesarean delivery (PMCD) in Japan

Case	1	2	3	4	5	6
Age, years	33	29	28	36	27	28
Gestational age, weeks	37	36	27	38	32	35
Site of occurrence	Ambulance	Clinic	Operating room	Ambulance	Operating room	Home
Underlying disease	Hemophagocytic syndrome (found at autopsy)	Hypokalemia	Twins, premature labor, pulmonary edema caused by magnesium formulation	Unknown	Twins, severe pre‐eclampsia, pulmonary edema	Unknown aortic dissection suspected
Initial ECG	PEA	Vf	pVT	Unknown	PEA	Unknown
CPA admission	9 min	30 min or more	Nosocomial incidence	20 min	Nosocomial incidence	28 min
CPA PMCD	41 min	51 min or more	Unknown	22 min	4 min	36 min
Admission PMCD	32 min	21 min	―	2 min	―	8 min
Resuscitation	Unsuccessful	Success	Success	Success	Success	Unsuccessful
VA‐ECMO	Tried to introduce but failed	Used	Did not use	Did not use	Did not use	Did not use
Hysterectomy	None	Done	None	None	None	None
Blood loss	6,700 mL	400 mL	Unknown	Unknown	2,150 mL	Unknown
Outcome of mother	Died in the emergency room	Survival to hospital discharge	Survival to hospital discharge	Died after 28 days	Survival to hospital discharge	Died in the emergency room
Outcome of baby	Death	Survival to hospital discharge	Unknown	Survived	Survival to hospital discharge	Unknown

CPA, cardiopulmonary arrest; ECG, electrocardiogram; PEA, pulseless electrical activity; pVT, pulseless ventricular tachycardia; VA‐ECMO, venoarterial extracorporeal membrane oxygenation; Vf, ventricular fibrillation.

## Conclusion

Perimortem cesarean delivery, which has been recommended by various international guidelines, is an effective strategy to rescue a pregnant patient and baby. However, stabilization of circulation using VA‐ECMO should be the top priority as it is extremely difficult to start PMCD within 4 min, and maintain the quality of CPR during PMCD. Moreover, the strategy of using DCS in PMCD is crucial to achieve effective hemostasis, as patients who need PMCD are critical. It is suggested that the combination of stabilization of circulation by VA‐ECMO before PMCD and PMCD using the damage control concept may be an excellent strategy for success of PMCD.

## Disclosure

Informed Consent: Written consent was obtained from the patient and their family to potentially publish their case. Conflict of Interest: None declared.

## References

[1] American Heart Association . 2010 American Heart Association Guidelines for cardiopulmonary resuscitation and emergency cardiovascular care. Circulation 2010; 122: S829–61. 20956228 10.1161/CIRCULATIONAHA.110.971069

[2] Katz V , Balderston K , DeFreest M . Perimortem cesarean delivery: were our assumptions correct? Am. J. Obstet. Gynecol. 2005; 192: 1916–20. 15970850 10.1016/j.ajog.2005.02.038

[3] Katz V , Dotters DJ , Droegemueller W , *et al* Perimortem caesarean delivery. Obstet. Gynecol. 1986; 68: 571–6. 3528956

[4] Department of Health, Welsh Office, Scottish Office Department of Health, Department of Health and Social Services, Northern Ireland . Why mothers die. Report on confidential enquires into maternal deaths in the United Kingdom 2000‐2002. London: The Stationery Office; 2004.

[5] Safar P , Brown T , Holtey W , *et al* Ventilation and circulation with closed chest cardiac massage in man. JAMA 1961; 176: 574–6. 13745343 10.1001/jama.1961.03040200010003

[6] Guidelines for cardiopulmonary resuscitation and emergency cardiac care . Emergency Cardiac Care Committee and Subcommittees, American Heart Association, part IV, special resuscitation situation. JAMA 1992; 268: 2242–50. 1404770

[7] European Resuscitation Council . European Resuscitation Council Guidelines for resuscitation 2015. Resuscitation 2015; 95: 148–201. 26477412 10.1016/j.resuscitation.2015.07.017

[8] Royal College of Obstetricians and Gynecologists . Maternal Collapse in Pregnancy and the Puerperium. Green‐top Guideline No. 56. London: Royal College of Obstetricians and Gynecologists; 2011. 10.1111/1471-0528.15995 31845507

[9] Einav S , Kaufman N , Sela HY , *et al* Maternal cardiac arrest and perimortem caesarean delivery: evidence or expert‐based? Resuscitation 2012; 83: 1191–200. 22613275 10.1016/j.resuscitation.2012.05.005

[10] Rotondo MF , Schwab CW , McGonigal MD , *et al* Damage control: an approach for improved survival in exsanguinating penetrating abdominal injury. J. Trauma 1993; 35: 375–82. 8371295

